# Supplementary material for: Paleobiological implications of chevron pathology in the sauropodomorph Plateosaurus trossingensis from the Upper Triassic of SW Germany
Source: PLoS One. 2024 Jul 31;19(7):e0306819. doi: 10.1371/journal.pone.0306819 (PMC11290664; doi:10.1371/journal.pone.0306819)
Supplement: S1 File — (DOCX) [file pone.0306819.s001.docx]

**Supporting information**

**S1 Table. Length in centimeters of chevrons of four specimens of *Plateosaurus trossingensis* from the Trossingen locality.**

| **Chevron** | **SMNS 13200** | **GPIT-PV-30784** | **SMNS 91298** | **SMNS 91306** |
| --- | --- | --- | --- | --- |
| **1** | 22.5 | 11.9 | 15.4 |  |
| **2** | 23.8 | 18.8 | 22.5 |  |
| **3** | 26.0 | 21.5 |  |  |
| **4** | 26.0 | 23.0 | 22.0 | 25.0 |
| **5** | 25.3 | 22.1 | 21.0 |  |
| **6** | 25.0 | 21.2 | 20.0 | 24.6 |
| **7** | 24.0 | 20.4 | 19.0 | 23.5 |
| **8** | 19.4 | 19.1 | 18.2 | 22.5 |
| **9** | 19.5 | 17.5 | 17.3 | 21.4 |
| **10** | 18.5 | 16.9 | 16.4 | 20.2 |
| **11** | 18.7 | 16.1 | 15.2 |  |
| **12** | 17.7 | 15.6 |  | 18.6 |
| **13** | 17.5 | 14.6 |  | 17.5 |
| **14** |  | 13.2 |  | 16.5 |
| **15** | 16.3 | 12.8 |  | 15.2 |
| **16** | 15.0 | 11.0 |  | 14.6 |
| **17** | 13.0 | 11.5 |  | 13.6 |
| **18** | 12.2 | 9.8 |  | 12.7 |
| **19** | 11.4 | 10.0 |  | 11.2 |
| **20** | 10.5 | 9.4 |  | 10.0 |
| **21** | 9.1 | 8.7 |  |  |
| **22** | 9.2 | 8.6 |  |  |
| **23** | 8.6 | 8.5 |  |  |
| **24** | 8.1 | 7.0 |  |  |
| **25** | 6.9 | 7.0 |  |  |
| **26** | 6.9 | 6.3 |  |  |
| **27** | 6.2 | 5.0 |  |  |
| **28** |  | 5.2 |  |  |
| **29** | 4.5 | 4.9 |  |  |
| **30** |  | 4.5 |  |  |
| **31** |  | 3.7 |  |  |
| **32** |  | 3.5 |  |  |

**S1 files. 3D model and µCT scans**

The µCT data and 3D models can be accessed on Figshare ([www.figshare.com](http://www.figshare.com)) with the DOI 10.6084/m9.figshare.26044132
