# Supplementary material for: Paleobiological implications of chevron pathology in the sauropodomorph Plateosaurus trossingensis from the Upper Triassic of SW Germany
Source: PLoS One. 2024 Jul 31;19(7):e0306819. doi: 10.1371/journal.pone.0306819 (PMC11290664; doi:10.1371/journal.pone.0306819)
Supplement: Attachment — Submitted filename: Rebuttal letter_Schaeffer_etal_.docx [file pone.0306819.s002.docx]

Dear editor,

Thank you for considering this manuscript for publication in *PLoS ONE*. We submit a revised version of the manuscript with ID PONE-D-24-09200 and entitled “**Paleobiological implications of chevron pathology in the sauropodomorph *Plateosaurus trossingensis* from the Upper Triassic of SW Germany**”. In this revised version we have addressed all the comments, questions and suggestions of the editor and reviewer, which were very useful and constructive. We have applied all of the requested changes and have modified a few other parts of the text to fix spelling and grammatical issues as well as an additional change in the discussion to strengthen the claims made (but without changing the message nor meaning of the previous version). All changes in the manuscript were done with the ‘track changes’ tool of Microsoft Word and both a manuscript with track changes as well as one without have been provided.

In the next pages of this letter we provide a response to all comments. We addressed all comments on a one by one basis. The comments of the reviewers are written in bold. Our answers are preceded by “**A:**” and are in blue.

We hope that the revised version of the manuscript addressed all issues commented on and fulfils the publication standard of *PLoS ONE*. We are looking forward to hearing back from you.

Yours sincerely,

Joep Schaeffer

Staatliches Museum für Naturkunde Stuttgart

Rosenstein 1, D-70191 Stuttgart, Germany

Email: [joep.schaeffer@smns-bw.de](mailto:joep.schaeffer@smns-bw.de)

Phone: +49 (0) 711 89 36 164

**Comments of the editor:**

**Remove from the text all citations that have not yet been accepted or published**

A. We have changed the citation of “Schaeffer (in review)” to “Schaeffer 2024”, as it was published last month. The reference is added to the list.

**Include number 2, 3 and 4 in figure 1. They are described in the text but not in the figure.**

A. This was a misunderstanding. We tried to refer to figures 1, 2, 3 and 4. We have adapted the figure citations in the text to be in line with the guideline of *PLoS ONE*.

**For the introduction, I advice the authors to check Pardo-Pérez et al., 2018, 2019 and 2020 papers on paleopathologies of ichthyosaurs, specially the last one regarding tail lessions: Pardo-Pérez, J., Kear, B., Maxwell, E.E. (2020). Skeletal pathologies track body plan evolution in ichthyosaurs. Scientific Reports. 10(1), 4206**

A. Thank you for the suggestion. We had already read these papers, and have now added Pardo-Pérez et al. 2020 to the list of used papers.

**Comments of the reviewer**

**I would like to have one or two lines, at the end of the introduction (before the geological setting), to tell me which are the specific objectives of the paper. "here we focus on two individuals [...] with three consecutives chevrons displaying pathologies"... for? how? What kind of analysis will you use? What kind of information do you need to provide? What kind of implications are you expecting? The introduction seems to end abruptly, and it might cause the reader to be a bit confused (at least, this is the feeling I had while going through it)**

A. Thank you for these comments. We fully agree with your opinion that the introduction ends too abrupt and is lacking a proper ending. We have revised and added a few sentences at the end of the introduction to make the objectives of the study more clear.

**Additional changes**

- As requested the Data repository was changed from Morphosource to Figshare (DOI 10.6084/m9.figshare.26044132)
- We have changed the Morphosource link to the permanent link where the supplementary 3D models and µCT scans can be downloaded.
- We have strengthened the suggestion made in lines 402-405 of the new version of the manuscript with track changes hidden by adding a comparison to other taxa within the same group and providing two additional citations.
- Several spelling and grammatical errors have been corrected throughout the manuscript.
